# Discovery of Intra-Abdominal Hemorrhage during Hernioplasty in a Patient Taking Rivaroxaban

**DOI:** 10.1155/2015/345716

**Published:** 2015-04-12

**Authors:** Harry Chow, Michael K. Ng

**Affiliations:** Saint John of God Hospital Ballarat, 101 Drummond Street North, Ballarat, VIC 3350, Australia

## Abstract

We discuss the first ever case of rivaroxaban causing major intra-abdominal hemorrhage found in a patient during emergency hernioplasty. The source of bleeding was not identified either intra- or postoperatively. This is on a background of treatment for deep vein thrombosis (DVT) with rivaroxaban, a complication that resulted from a total knee arthroplasty performed four weeks prior. Rivaroxaban is a new generation of anticoagulants that directly inhibits factor Xa and is used for DVT treatment in major orthopaedic surgery. Here we discuss the major side effects of rivaroxaban, namely, the increased risk of major bleeding as well as the irreversibility of this anticoagulant, should such bleeding occur. We advise caution in the use of rivaroxaban even in patients that are at low risk of bleeding given the discovery of hemorrhage as presented in this case.

## 1. Case Presentation

A 72-year-old Caucasian female presented to the emergency department with two days of nausea and vomiting and on examination a large palpable left iliac fossa mass. The patient however described normal bowel actions over the preceding two days and was pain-free.

Her past medical history included a recent total knee arthroplasty (TKA) four weeks previously and the commencement of rivaroxaban, a direct Factor Xa inhibitor, a new anticoagulant which had been used to treat deep vein thrombosis (DVT) which had occurred postoperatively on the knee replacement side. Other comorbidities included hypertension, hypercholesterolaemia, and osteoarthritis which she was on medication for.

**Figure 1:**
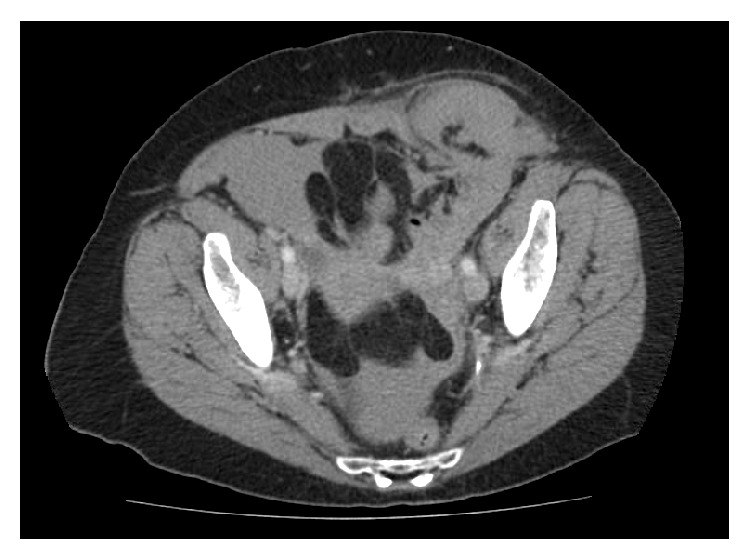
Preoperative noncontrast abdominal CT scan showing a large left indirect inguinal hernia sac.

Prior to surgery, a noncontrast computed tomography (CT) scan of the abdomen and pelvis showed a large amount of free fluid, around the liver and descending colon, within the pouch of Douglas, and bilaterally in the lower pelvis, including within a large left indirect inguinal hernia sac.  Figure 1 shows a snapshot of the CT scan performed showing the large fluid-filled indirect inguinal hernia sac. The presence of a fluid-filled level within the hernia sac and free fluid within abdomen created concerns of a strangulated hernia, and the patient was scheduled for an urgent open left inguinal hernioplasty.

Her preoperative status was within normal physiological parameters (blood pressure 132/70, heart rate 82 beats/minute, and oxygen saturation 100% in room air), hemodynamically stable with a hemoglobin level of 10.6 g/dL prior to surgery. Rivaroxaban was ceased prior to surgery.

At surgery, a left inguinal incision was performed, with exploration of the inguinal canal. A large indirect hernia sac was identified with a firm consistency with its contents. Opening of the sac revealed a large volume of blood and clot. The bowel was otherwise viable with no sign of perforation or other intra-abdominal contamination. A left inguinal hernioplasty was performed with a standard Lichtenstein repair.

She spent the subsequent two days as an inpatient under observation. She did not require blood transfusions during this period. Her hemoglobin levels over these two days fell to 93 g/dL and subsequently to 83 g/dL the day after. She was subsequently discharged home without further event.

Postoperative review at two weeks did not show any abnormalities and subsequent intravenous contrast CT scan of her abdomen at 3 weeks was performed to ensure no obvious intraperitoneal cause for the bleeding. There was no evidence of residual hematoma, or any evidence of intra-abdominal lesion which may have accounted for unexplained intra-abdominal bleeding.

## 2. Discussion

A thorough search of the literature shows this is a unique case in which a patient on rivaroxaban developed a potentially life-threatening intra-abdominal hemorrhage. Within her history the patient had no comorbidities or pathology identified on imaging that would indicate a likely source of the bleeding. The only contributor had been the use of rivaroxaban, a direct Factor Xa inhibitor, one of a number of new anticoagulants targeted for use in treatment and prevention of venous thromboembolism (VTE) following major orthopaedic surgery [1].

The risk of DVT development in major orthopaedic surgery, in this case TKA, is 40–60% without adequate prophylaxis [2]. In cases where symptomatic VTE has occurred, as was the case in this patient, the standard treatment has been initial bridging heparin, overlapping with the addition of a vitamin K antagonist such as warfarin. The duration of this treatment is typically twelve months, with the reduction of VTE recurrence from 25% to 3% and 5–10% during the first year of treatment cessation [3]. The effectiveness of the current standard therapy is hampered by therapy duration and the limitations of the current agents. Heparin requires daily parenteral administration whereas warfarin's low therapeutic window requires daily laboratory monitoring and constant dose adjustment [4]. This burden greatly affects patient compliance which is why there is an increased interest in viable alternatives such as rivaroxaban.

Rivaroxaban has been shown in studies to have the same efficacy as or superior efficacy to warfarin in terms of primary and systemic VTE treatment for major orthopaedic surgery without the limitation of its predecessors [5]. It claims to offer rapid oral absorption and predictable levels within the body not requiring daily monitoring [6]. The obvious benefits of rivaroxaban have overshadowed its rare but potentially life-threatening complications. There have now been an increasing number of studies in the literature indicating the side effect profile of rivaroxaban which has led many to suggest caution in its use over standard DVT treatment. The most major side effects of rivaroxaban have been both bleeding and irreversibility [1].

Many of these studies highlight that the bleeding complications of rivaroxaban relate to its use in major orthopaedic surgery, particularly total hip and knee arthroplasty. These studies have shown a significantly increased risk of nonmajor and major hemorrhage, 5.4% and 0.7%, respectively, with rivaroxaban use [5]. This has been particularly with early postoperative complications such as increased risk of surgical site hemorrhage requiring transfusion and revision surgery [7]. Others have noted that there are increased wound complications in patients on rivaroxaban, with significant increases in hematoma formation following arthroplasty [8].

The other major area where complications from rivaroxaban use have arisen is bleeding into the gastrointestinal system. A meta-analysis of the literature showed that there was a moderate but significantly higher risk of gastrointestinal bleeding in patients taking rivaroxaban compared to those on standard VTE treatment [9]. One case report in particular described a patient who had a severe life-threatening, gastrointestinal hemorrhage after TKA related to rivaroxaban, which required multiple transfusions of blood [10].

There is currently no ability to fully reverse the effects of rivaroxaban should a catastrophic bleed occur [11]. At this point in time, studies in rats have only shown partial reversal of rivaroxaban with recombinant activated factor VII [12]. This is compounded by the pharmacokinetics of rivaroxaban which has a rapid onset of action taking 2-3 hours to reach peak plasma concentration and a half-life of 8–10 hours [13]. Additionally, the clearance of rivaroxaban is greatly impaired in patients with reduced renal function and has not been recommended for use in patients with severe impairment [14]. Given that patients receiving TKA often have these comorbidities, it is dangerous to note that should hemorrhaging occur there are no clear measures to counteract rivaroxaban's effects.

This clinical case presents a patient on rivaroxaban that developed a potentially life-threatening, intra-abdominal hemorrhage, discovered fortuitously only upon presentation as a large acute inguinal hernia. Had there been no inguinal hernia, the presence of intra-abdominal bleeding may well have remained unnoticed for much longer, with potentially dire consequences. The patient had no previous history of bleeding or risk factors that would indicate an identifiable source of hemorrhage into the abdominal cavity. The use of rivaroxaban for treatment of her DVT after TKA points to the only contributing factor toward the bleeding in an otherwise low-risk patient. Given that this patient had intra-abdominal bleeding whilst on anticoagulation, had bleeding continued, more aggressive surgical exploration would have been required to find a potential bleeding source. It was fortuitous therefore that bleeding ceased, given that there was no recognisable source, as it might have impacted negatively on her outcome.

## 3. Conclusion

Rivaroxaban, whilst having demonstrated efficacy in VTE treatment, can have devastating, life-threatening side effects, especially massive intra-abdominal hemorrhage. Also, as this drug is currently not reversible, in the acute situation, this may potentially not be a salvageable situation. Caution should be exercised in the use of rivaroxaban even in patients that are not deemed to be of high risk, due to the possibility of spontaneous bleeding as presented in this case.
